# The impact of completing upper secondary education - a multi-state model for work, education and health in young men

**DOI:** 10.1186/s12889-018-5420-y

**Published:** 2018-04-27

**Authors:** Rune Hoff, Karina Corbett, Ingrid S. Mehlum, Ferdinand A. Mohn, Petter Kristensen, Therese N. Hanvold, Jon M. Gran

**Affiliations:** 1 Oslo Centre for Biostatistics and Epidemiology, Department of Biostatistics, University of Oslo, Oslo, Norway; 2 National Institute of Occupational Health, Oslo, Norway; 3 Institute of Health and Society, University of Oslo, Oslo, Norway; 4 Oslo Centre for Biostatistics and Epidemiology, Oslo University Hospital, Oslo, Norway

**Keywords:** Multi-state models, Upper secondary education, Dropout, Work participation, Sickness absence, Unemployment, Disability

## Abstract

**Background:**

Completing upper secondary education is associated with higher work participation and less health-related absence from work. Although these outcomes are closely interrelated, most studies focus on single outcomes, using cross-sectional designs or short follow-up periods. As such, there is limited knowledge of the long-term outcomes, and how paths for completers and non-completers unfold over time. In this paper, we use multi-state models for time-to-event data to assess the long-term effects of completing upper secondary education on employment, tertiary education, sick leave, and disability pension over twelve and a half years for young men.

**Methods:**

Baseline covariates and twelve and a half years of follow-up data on employment, tertiary education, sick leave and disability pension were obtained from national registries for all males born in Norway between 1971 and 1976 (n =184951). The effects of completing upper secondary education (by age 23) were analysed in a multi-state framework, adjusting for both individual and family level confounders. All analyses were done separately for general studies and vocational tracks.

**Results:**

Completers do better on a range of outcomes compared to non-completers, for both fields of upper secondary education, but effects of completion change over time. The largest changes are for tertiary education and work, with the probability of work increasing reciprocally to the probability of education. Vocational students are quicker to transfer to the labour market, but tend to have more unemployment, sick leave and disability, and the absolute effects of completion on these outcomes are largest for vocational tracks. However, the relative effects of completion are larger for general studies.

**Conclusion:**

Completing upper secondary education increases long-term work participation and lowers health-related absence for young men, but effects diminish over time. Studies that have used shorter follow-up periods could be overstating the negative effects of dropout on labour market participation. Multi-state models are well suited to analyse data on work, education and health-related absence, and can be useful in understanding the dynamic aspects of these outcomes.

## Background

Completing upper secondary education [1] is known to be associated with higher work participation and reduced health-related absence in young adulthood [2–7]. The associations remain after adjusting for known predictors of completing upper secondary education and relevant outcomes [3, 4]. However, most studies focus on single outcomes, using cross-sectional designs or short follow-up time, for instance looking at subjects’ first period of long-term sick leave or time to disability. Such studies give results that are easy to communicate, but the choice of outcome measure, and the time of measurement, can often seem arbitrary. If detailed individual follow-up data is available over a longer period, we may consider several outcomes, which together constitute a continuous outcome process, where individuals move back and forth between different types of states over time. By utilizing the individuals’ full outcome trajectories, we can analyse how paths for completers and non-completers unfold over a longer period and estimate the time-varying effects of having completed upper secondary education on all outcomes together.

Dropout and health disparities are very closely linked [2–4, 8], and some have argued that school dropout is essentially a public health issue [8]. Non-completers may be at a disadvantage when trying to enter the job market, they might take jobs that are more taxing on health, they might have higher chances of being laid off, and the lack of a diploma may prevent them from entering colleges and universities. However, dropping out, or conversely, completing different types of education, do not happen by coincidence. Rather it is a process starting in early childhood, associated with early home environment, quality of caregiving, socioeconomic position (SEP), intelligence, behaviour problems, peer relations, and parent involvement [9]. Students differ in background skills and several other traits. A study from 2012 examined 110 so-called dropout indicators and found that the three best predictors of completion were growth in mathematics test scores from grades 7-12, growth in GPA (Grade Point Average) from grades 9-12 and level of school engagement [10]. In addition to academic skills, mental health problems are strongly associated with increased risk of dropout[11–14]. Others have reported that completion rates to some degree are explained by labour market characteristics in the corresponding residential region [7, 15].

Predictors of non-completion and completion may also be linked to the outcomes we consider, hence making them confounders. For instance, Plomin and Deary [16] concluded that intelligence is one of the best predictors of important life outcomes, including occupation, mental and physical health. Other studies find a clear association between intelligence and adolescent SEP and adult educational attainment. In a paper from 2009, analysing a cohort of males born in Norway between 1967–1971 (n =160 914), years of education at age 28 was strongly associated with intelligence test score and parental education, while parental income had a smaller influence [17]. Other studies suggest that disability is to some degree “inherited” from parents down to children [4]. A naive unadjusted analysis is likely to yield a biased comparison of the exposures non-completion and completion. However, if sufficient covariate information is available, we can adjust for confounding variables such as these to reduce the bias.

Statistics Norway produces official statistics and collects detailed individual follow-up data on work participation, education and health-related absence for all Norwegian citizens. Recently, several papers have demonstrated that multi-state models are suitable for analysing data of this type [18–24]. In the multi-state framework, hazard-based methods for survival data can be used to model transitions, for instance using Cox proportional hazards models, Aalen additive hazards models or the Nelson-Aalen estimator [25]. For a detailed introduction to multi-state models for time-to-event outcomes, see for instance [26–30].

The Norwegian education system consists of mandatory primary education [1] that lasts for seven years (six at the time of our study population) followed by three years of mandatory lower secondary education. After graduating, usually the year individuals turn 16, students may enrol in upper secondary education or discontinue further education. More than 95% of the youths choose to continue. Upper secondary education in Norway consists of two distinct fields; general studies and vocational tracks. General studies are geared towards tertiary education [1] in college or university; vocational tracks are geared towards specific trades. One may also obtain admission right to colleges and universities from a supplementary year after vocational tracks. Alternatively, one can obtain admission right at the age of 23 by having 5 years of education or a combination of specific types of experience (employment/volunteering/folk high school/military duty) and 1 year of upper secondary education. In other words, one does not need to have completed general studies for admittance into tertiary education. Normal time spent for general studies is 3 years. For vocational tracks, a duration of 3-5 years is normal, 2 years at school followed by two years as an apprentice is most common.

Previous studies on consequences of non-completion or completion have typically had a relatively short follow-up time, focused on isolated outcomes or not done separate analyses for vocational tracks and general studies [2, 3, 5–7]. However, the two fields are arguably not comparable in terms of learning outcome and type of students, so the effects could be quite different [31]. Thus, there is a need to evaluate long-term outcomes for both fields.

In this paper, we analyse the long-term effects of completing upper secondary education by the age of 23 on the outcomes work, unemployment, tertiary education, sick leave and disability pension over a twelve and a half years period. The use of exactly twelve and a half years of follow-up, was a consequence of the inclusion age and the maximum follow-up time for people in the birth year cohorts included in our data. In our analyses, we consider every individual’s outcomes of work, unemployment, education (tertiary), sick leave and disability continuously throughout the follow-up period. To make the comparison of completers and non-completers as unbiased as possible, we adjust for a wide set of baseline confounders. As analyses are done separately for general studies and vocational tracks, we illustrate how outcomes and effects of completion unfold over time within each field.

## Methods

### Data sources

The data material comes from a cohort consisting of all males born in Norway between 1971 and 1976 (n =184 951). The gender restriction allowed for using military conscript data (IQ, BMI, military eligibility check). Baseline characteristics and data on work participation, education and health-related absence are available from several national population-wide registries. Personal identification numbers allowed for linking within study subjects and between subjects and parents across several registries: Statistics Norway’s events database on employment and welfare, FD-Trygd, The National Education Database, The Armed Forces Personnel Data Base and the registries of the Norwegian Labour and Welfare Administration.

### Study population

**Fig. 1:**
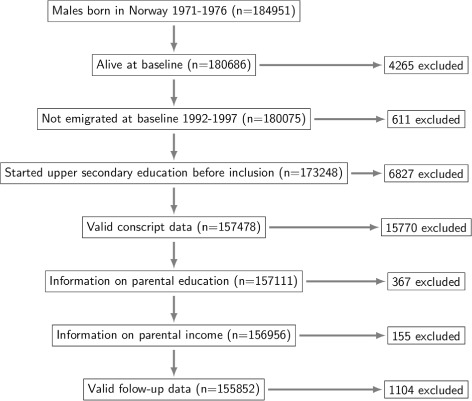
Flow chart for exclusion process. Showing the number of individuals included and excluded at each step of the exclusion process

All individuals were included in the study from the 1st of July the year they turned 23 (1994–1999) and followed for 12.5 years, until 31st of December (2006–2011). Individuals who emigrated before the study start were completely removed from the dataset. Emigration occurring after the start of follow-up resulted in temporary removal from the dataset, given that the individual returned. This is also how paternal leave (paid leave a father takes off work at the birth or adoption of a child) was handled. Furthermore, we only included those who had started an upper secondary education program before the year they turned 21 (96.2% of the alive population). Individuals also needed complete conscript data, information on parental SEP (education and income) and valid follow-up data to be included. Invalid follow-up data in our study, could for instance be to start at disability pension or only being observed in paternal leave. After applying exclusion criteria, we ended up with a sub-cohort of 155 852 individuals for analysis; 62803 students of general studies and 93049 students in vocational tracks. See Fig. 1 for an overview of the study selection and exclusion process.

Throughout the follow-up period we had individual state trajectories for every subject. The register databases from where we create state trajectories exhibit a high degree of completeness. More so, near 100 percent of the individuals in the study (after applying the exclusion criteria listed in Fig. 1) were under observation at the inclusion date. There is a small number of individuals not registered in any state at that time who enters observation shortly after. At the end of follow-up, 95 percent were still under observation, and it was assumed that missingness was not informative, although this could be investigated and, if necessary, alleviated with inverse probability of censoring weights [32, 33].

### Multi-state model for work participation, health-related absence and education

**Fig. 2:**
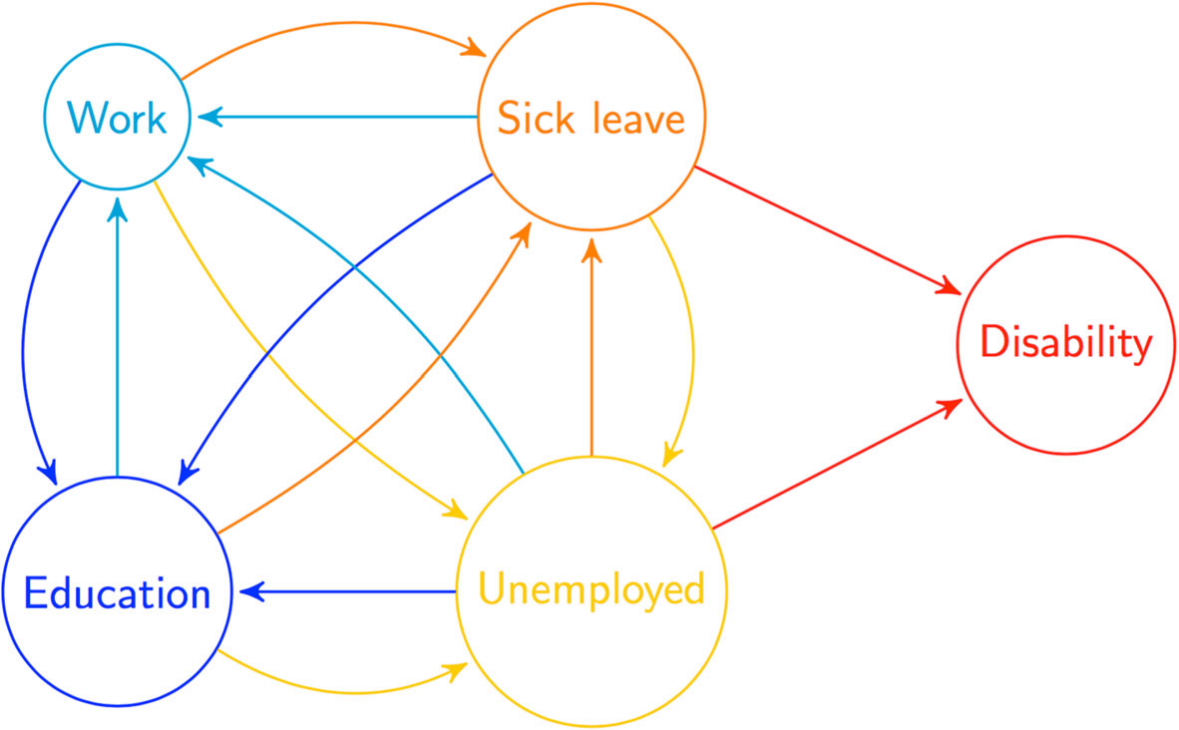
States. The states in the multi-state model with arrows representing possible transitions

To assess the effects of completion, we fitted a multi-state model for analysing time-to-event outcomes. An event is here a transition between different states. These states were *work*, *unemployment*, *education* (tertiary), *sick leave* and *disability*. The states and all possible transitions between them are summarised in Fig. 2. In some cases e.g. for students working part time or individuals on partial sick leave while working part-time, a decision had to be made regarding which state they belonged to. To handle such issues and keep the number of states manageable, states were given different precedence when individuals qualified to more than one state at the same time. In order of decreasing precedence we prioritized disability, sick leave, unemployment, work over lastly, education. An exception was made whenever work resulted in yearly income less than 2G; 1G being defined by the Norwegian Labour and Welfare Administration to calculate various types of welfare pensions (1G as of 1. May 2017 equals 93 634 NOK). In the case of income less than 2G, education would have precedence over work. 2G was used to separate between students and workers, as it is slightly over the maximum allowed income for entitlement to full student loan and grant. This ensured that most students working part-time were considered students (education state), while full-time employees, earning more than 2G, could be attending educational courses and still be considered workers (work state). Only a few observations were made of people leaving the disability state during follow-up, we therefore considered disability as an absorbing state. Additionally, we did not consider transitions directly into disability from work and education due to the rarity of such events, and because such transitions would normally not follow regulatory laws concerning disability allowance and are likely to be purely administrative artefacts.

### Exposure variable

We considered our exposure *completing upper secondary education* as having obtained a degree by July the 1st the year the subject turned 23. Individuals who did not finish within this time frame were either dropouts or had delayed completion, hereafter referred to as non-completers. The information on obtained degrees came from The National Education Database.

### Baseline covariates

Through various administrative registers we had access to numerous covariates at individual level. The variables included in our analysis fell into three categories: 1) *family background variables*: parental education, income, disability history and mother’s marital status – together accounting for an individual’s background and upbringing. 2) *Individual variables*: IQ, BMI, military eligibility check (mental and physical health) and childhood chronic disease history – accounting for cognitive ability and health. 3) *Societal/contextual variables*: regional unemployment rate and year of birth. Year of birth is included to adjust for an increasing completion rate in later birth cohorts and economic cycles of recession and boom periods. Summarising statistics of the covariates are shown in Table 3. Reference categories used in the Cox models are marked by (*) in the table.

### Fitting multi-state models

To estimate state probabilities, we first introduce some standard multi-state notation. Let *X*(*t*) denote the state of an individual at time *t*. The transition probability matrix **P**(*s*,*t*) shall consist of elements *P*_*hj*_(*s*,*t*)=*P*(*X*(*t*)=*j*|*X*(*s*)=*h*), which represent the probability of going from state *h* to state *j* during the time interval (*s*,*t*]. By assuming that the model is Markov, which is an assumption we discuss later, this transition probability matrix can be estimated with the matrix product-formula:

1 $$  \hat{\mathbf{P}}(s,t) = \underset{u \in (s,t]}{\prod} (\mathbf{I} + d \hat{\mathbf{A}}(u)),  $$

where $\hat {\mathbf {A}}(u)$ is the cumulative transition intensity matrix at time *u* [34]. The matrices $\hat {\mathbf {A}}(u)$ can for instance be identified with transition specific Nelson-Aalen estimates.

If we model transitions by conditioning on baseline covariates *Z*, the matrix product-formula becomes:

2 $$  \hat{\mathbf{P}}_{Z}(s,t) = \underset{u \in (s,t]}{\prod} \left(\mathbf{I} + d \hat{\mathbf{A}}_{Z}(u)\right),  $$

Now $\hat {\mathbf {A}}_{Z}(u)$ is the conditional cumulative transition intensity matrix at time *u*. The elements of $\hat {\mathbf {A}}_{Z}(u)$ can, for example, be estimated from Cox proportional hazards models together with a non-parametric estimator for the baseline hazard [34].

Given the transition probability matrix, we can calculate the probability of being in state *j* at time *t* when starting in state *h* by *P*_*hj*_(0,*t*). More generally, the probability of being in state *j* at time *t*, so-called state probabilities, can be calculated by:

3 $$  \hat{P}(X(t)=j) = \sum_{k} \hat{P}_{kj}(0,t) \cdot \hat{P}(X(0)=k).  $$

Without covariates, *P*(*X*(0)=*k*) is calculated by the proportion of subjects entering the study in state *k* at time equal zero. In covariate adjusted models, we may need to estimate *P*(*X*(0)=*k*|*Z*) – for instance by predicting from a multinomial logistic regression using starting state as outcome.

The multi-state models in our analyses rely on a Markov assumption, which requires that the instantaneous risk of transition to any other state, only depends on the current state and not the state history. With data on unemployment and health-related absence, which abides regulatory laws regarding maximum allowed length of stay, the Markov assumption will typically be violated for some types of transitions. Deviations from the assumption could be explored and perhaps alleviated through semi-Markov models [26, 27, 29, 30]. However, when focusing on estimating state probabilities, the estimator in Eq. 3 has been proven to be consistent, also in the presence of violations to the Markov assumption [35].

### Inverse probability weighted multi-state models

We can adjust for covariates by fitting a multivariate hazard model, e.g. by Cox regression, for each possible transition in the multi-state model. However, when calculating state probabilities, we must then explicitly specify values for all covariates. It is impractical and often infeasible to do such calculations for all covariate patterns. When the aim is to adjust for confounding when identifying the effect of a main exposure, a multivariate regression approach will also not give such an effect directly. A better alternative is then to estimate the *average effect* of the exposure over all observed covariate patterns in the population using inverse probability of treatment weighting (IPTW) [36]. In order to do this, we estimate each subject’s probability of exposure given covariates. The idea is then to weight all observations with the corresponding individual’s inverse probability of treatment. Before weighting, completion and non-completion are unevenly spread over different covariate patterns, while in the weighted dataset both exposures are balanced (equally represented) across the patterns. This means completion and non-completion can be compared without adjusting for other baseline covariates.

To estimate the weights, we fitted a logistic regression model for completion versus non-completion, which was used to calculate probabilities of each individual’s exposure based on their covariates. After applying the weights, the only covariate that remains to be controlled for is the main exposure. This means we may predict state probabilities for completion and non-completion using either weighted univariate hazard regression models that only includes the exposure, or weighted Nelson-Aalen estimates for transition hazards in each exposure group separately.

Interpreting the effect from the IPTW analysis as the marginal effect of completion in the population, depends on various model and causal assumptions [18]. The most central among the latter assumptions, is the one of no unmeasured confounding; that we sufficiently adjust for all common causes of completing upper secondary education and later states. Furthermore, there must be a positive probability of completion and non-completion for all observed covariate values and consistency of the exposure [37, 38]. In addition, the model for estimating the weights must also be correctly specified.

### Technical remarks on model fitting

All analyses were done in R [39] version 3.4.1 using the survival- and mstate libraries [34]. Note that fitting multivariate Cox models for multi-state data of this magnitude and subsequently applying the matrix-product formula are computationally intensive tasks and could be problematic on standard computers and laptops. This work was partly performed on the Abel Cluster, owned by the University of Oslo and the Norwegian Metacenter for High Performance Computing (NOTUR), and operated by the Department for Research Computing at USIT, the University of Oslo IT Department [40].

The inverse probability weighted analysis, on the other hand, is more manageable on a regular computer. This is due to the fact that the logistic model is quick to optimise with an iterative reweighted least squares algorithm. The subsequent multi-state analysis can either be based on a univariate weighted hazard regression model or weighted Nelson-Aalen estimates for each exposure group, which is considerably less demanding in terms of computing power than a multivariate model.

## Results

### Unadjusted analysis

**Fig. 3:**
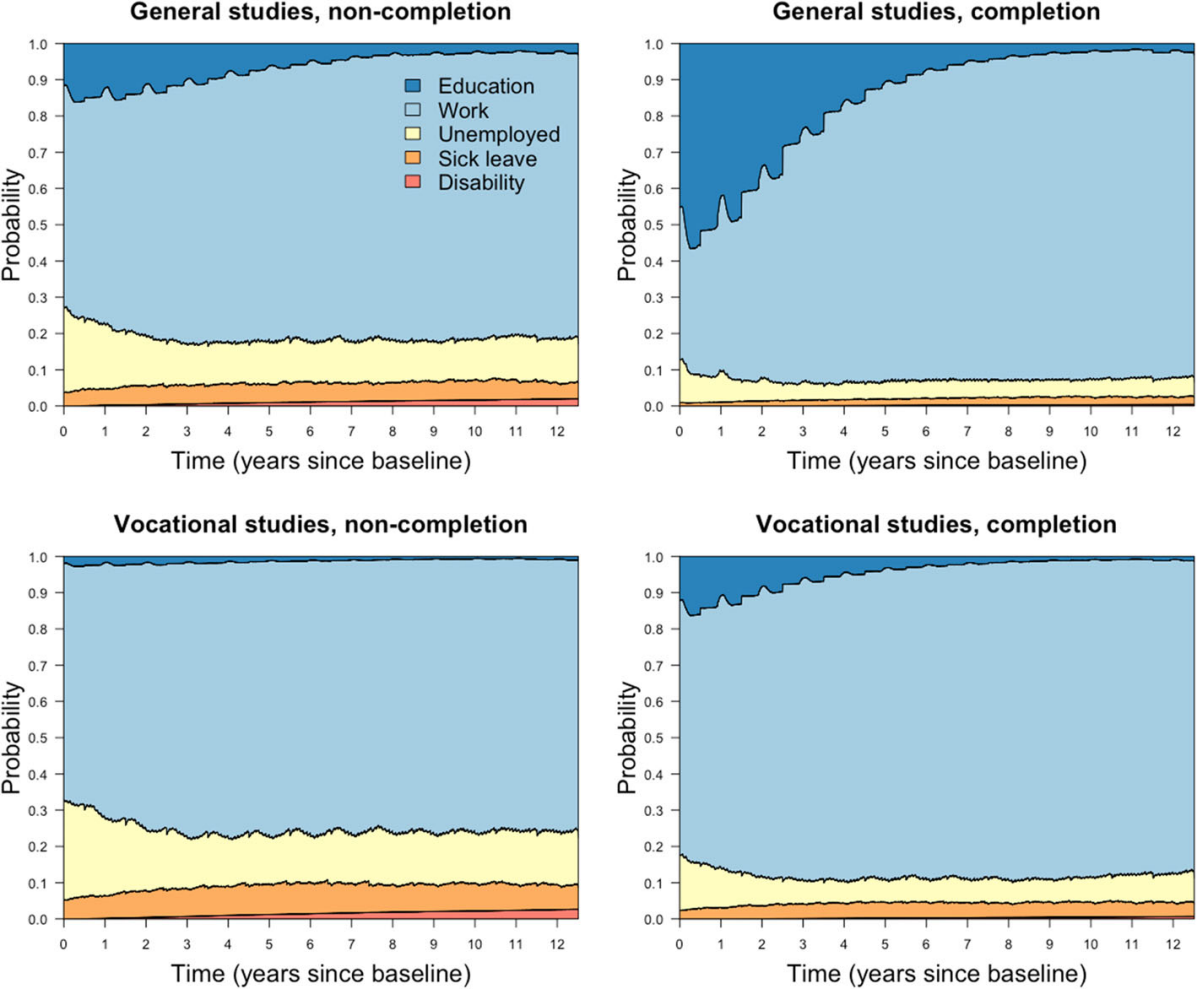
Unadjusted analysis. Unadjusted state occupation probabilities

The unadjusted effects of completion were analysed separately for general studies and vocational tracks by fitting transition specific Nelson-Aalen estimators, which were used to calculate state occupation probabilities, displayed in Fig. 3.

The plots show how the probability of each state changes over time. Many features are recurring, such as unemployment being at its highest early on, and gradually decreasing the first three years before stabilizing. Similarly, sick leave stabilises after around three years, but contrary to unemployment, this follows a gradual increase the first three years. Overall, general studies are associated with lower probabilities of unemployment, sick leave and disability compared to vocational tracks, and completers have lower probabilities of these states than non-completers. Students in the two fields of upper secondary education, as well as completers and non-completers, differ considerably when it comes to tertiary education early in the follow-up period. The ones most likely to be in education are those who completed general studies, while non-completers in vocational tracks are least likely to be in education. During the first 6 months, the mean percentages in education were the following; general studies non-completion: 14.7, vocational tracks non-completion: 2.4, general studies completion: 54.9, vocational tracks completion: 14.9. We notice a steep fall in the probability of education the first three years for completers of general studies, which can be seen in connection with transfers into work. As we move towards the end of follow-up and people finish studying, only small proportions are still in education across all groups.

### Cox regression

State transitions were analysed with three different types of Cox regression models. A univariate model that only included the main exposure; an inverse probability weighted univariate model and a multivariate model where main exposure and baseline covariates were included. The point estimates from a Cox analysis fail to illustrate the time aspect, but they are valuable in the sense that they say something about how exposure and covariates (if included) act on the transitions. In the multivariate model there are 336 parameters (24 for each of the 14 transitions), so we only included exposure effects in Table 1 (general studies) and Table 2 (vocational tracks) where we also show 95% confidence intervals for the estimates (robust variance used in the weighted analyses). The estimates are transition hazard ratios (HR) between completion and non-completion (reference non-completion). On any time point, the hazard can be thought of as the probability of transition within a short time-interval. Looking at the first three rows in the first column of Table 1, we find that completion of general studies is associated with roughly half the hazard of transition from work to unemployment (HR =0.53) or sick leave (HR =0.471), and more than double hazard of transition from work to education (HR =2.174) compared to non-completion. If we instead look at the second and third column, the point estimates are closer to 1 (1 means no effect), which means that the associations are somewhat reduced when we adjust for covariates.

**Table 1:** General studies, effect of completion, Cox regressions for transitions. The coefficients are hazard ratio estimates with lower and upper 95% confidence limits. Reference non-completion

	Univariate Cox	Multivariate Cox	Weighted univariate Cox
Work to Unemp	0.530 (0.519, 0.542)	0.608 (0.594, 0.622)	0.607 (0.590, 0.624)
Work to Sick leave	0.471 (0.462, 0.481)	0.570 (0.558, 0.582)	0.565 (0.551, 0.580)
Work to Education	2.174 (2.101, 2.249)	1.905 (1.840, 1.973)	1.693 (1.625, 1.763)
Unemp to Work	1.281 (1.256, 1.306)	1.191 (1.167, 1.216)	1.201 (1.165, 1.238)
Unemp to Sick leave	0.637 (0.606, 0.670)	0.736 (0.697, 0.778)	0.731 (0.688, 0.776)
Unemp to Education	3.277 (3.103, 3.462)	2.593 (2.450, 2.743)	2.214 (2.075, 2.361)
Unemp to Disability	0.454 (0.342, 0.603)	0.606 (0.441, 0.834)	0.554 (0.395, 0.777)
Sick leave to Work	1.401 (1.371, 1.432)	1.298 (1.269, 1.328)	1.314 (1.262, 1.369)
Sick leave to Unemp	0.859 (0.827, 0.893)	0.891 (0.855, 0.927)	0.903 (0.864, 0.945)
Sick leave to Education	4.881 (4.088, 5.828)	3.600 (3.005, 4.314)	3.317 (2.705, 4.068)
Sick leave to Disability	0.743 (0.561, 0.984)	0.929 (0.681, 1.267)	0.940 (0.685, 1.291)
Education to Work	1.115 (1.080, 1.150)	1.123 (1.089, 1.160)	1.136 (1.092, 1.181)
Education to Unemp	0.867 (0.817, 0.919)	0.898 (0.846, 0.953)	0.883 (0.820, 0.951)
Education to Sick leave	0.784 (0.654, 0.940)	0.837 (0.697, 1.006)	0.858 (0.693, 1.062)

**Table 2:** Vocational tracks, effect of completion, Cox regressions for transition events. The coefficients are hazard ratio estimates with lower and upper 95% confidence limits. Reference non-completion

	Univariate Cox	Multivariate Cox	Weighted univariate Cox
Work to Unemp	0.559 (0.552, 0.566)	0.626 (0.617, 0.634)	0.638 (0.628, 0.648)
Work to Sick leave	0.602 (0.596, 0.608)	0.686 (0.678, 0.693)	0.698 (0.689, 0.706)
Work to Education	2.632 (2.539, 2.729)	2.079 (2.004, 2.158)	1.968 (1.893, 2.046)
Unemp to Work	1.317 (1.302, 1.332)	1.253 (1.238, 1.268)	1.262 (1.240, 1.283)
Unemp to Sick leave	0.808 (0.788, 0.829)	0.879 (0.856, 0.903)	0.886 (0.861, 0.912)
Unemp to Education	4.401 (4.165, 4.651)	3.037 (2.868, 3.216)	2.982 (2.811, 3.163)
Unemp to Disability	0.498 (0.417, 0.596)	0.634 (0.526, 0.764)	0.640 (0.524, 0.780)
Sick leave to Work	1.302 (1.288, 1.316)	1.234 (1.220, 1.248)	1.235 (1.211, 1.260)
Sick leave to Unemp	0.892 (0.875, 0.910)	0.896 (0.878, 0.914)	0.893 (0.874, 0.912)
Sick leave to Education	3.324 (2.821, 3.916)	2.427 (2.048, 2.875)	2.335 (1.964, 2.775)
Sick leave to Disability	0.529 (0.440, 0.635)	0.672 (0.555, 0.813)	0.724 (0.590, 0.889)
Education to Work	1.094 (1.057, 1.132)	1.099 (1.062, 1.138)	1.114 (1.072, 1.158)
Education to Unemp	0.766 (0.721, 0.814)	0.803 (0.755, 0.854)	0.819 (0.764, 0.878)
Education to Sick leave	0.678 (0.567, 0.811)	0.727 (0.606, 0.872)	0.789 (0.650, 0.957)

**Fig. 4:**
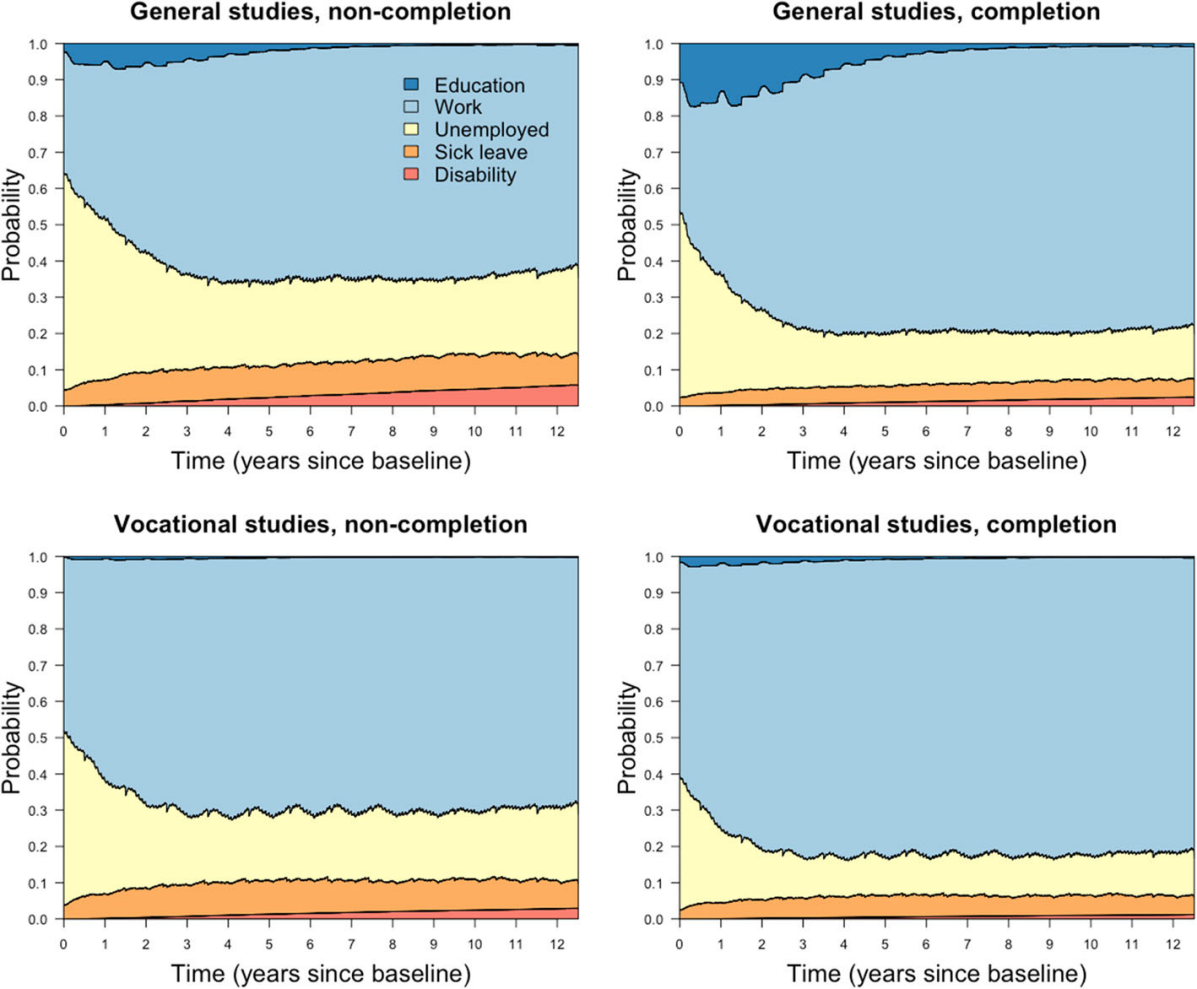
Cox regression. State occupation probabilities, unfavourable covariates

From the fitted Cox models, it is possible to calculate state probabilities for completion and non-completion while specifying values for the baseline covariates. We considered two different covariate patterns. In the first we specified typically *unfavourable* covariate values with IQ: 1-3, parental education: lower secondary education and parental income: less than 60% of median. The second was considered *favourable* with IQ: 7-9, parental education: university and parental income: more than 140% of median. In both cases, remaining covariates were set to reference level (indicated by a * in Table 3. The state probabilities for the first covariate combination are displayed in Fig. 4. Recall that in the unadjusted analysis, students in vocational tracks did worse for unemployment, sick leave and disability compared to general studies. Here, with unfavourable covariates, the situation is the opposite.

**Table 3:** Description statistics of covariates for the final cohort (n = 155852)

Covariate	Total number (%)		
	or mean (sd)	Completion, general	Completion, vocational
General studies			
Non-completion (*)	10461 (17%)		
Completion	52200 (83%)		
Vocational tracks			
Non-completion (*)	44927 (48%)		
Completion	47824 (52%)		
Year of birth			
1971 (*)	28361 (18%)	83%	46%
1972	28249 (18%)	83%	49%
1973	25117 (16%)	83%	51%
1974	25141 (16%)	84%	54%
1975	25050 (16%)	83%	56%
1976	23934 (16%)	85%	57%
Parental education			
Lower secondary education	17445 (11%)	68%	39%
Upper secondary education(*)	93824 (60%)	80%	52%
University	44583 (29%)	89%	63%
Parental income (% of median)			
Less than 60%	19018 (12%)	71%	40%
60-100%	53220 (34%)	80%	49%
100-140% (*)	60798 (39%)	85%	56%
Above 140%	22816 (15%)	89%	64%
Parental history of disability			
No (*)	109905 (71%)	85%	55%
Yes	45947 (29%)	78%	45%
Marital status mum			
Married (*)	116774 (75%)	86%	55%
Not married	2566 (2%)	69%	38%
Separated (subject 18)	23229 (15%)	74%	40%
Other	13283 (8%)	77%	45%
Childhood chronic disease benefit			
None (*)	154204 (99%)	83%	52%
Basic	747 (0.5%)	69%	39%
Attendance benefit	901 (0.5%)	72%	38%
Conscript IQ (stanine)			
1-3	24577 (16%)	47%	33%
4-6 (*)	91020 (58%)	79%	55%
7-9	40255 (26%)	90%	70%
Conscript BMI			
< 18.5	8224 (5%)	83%	47%
18.5 - 25 (*)	123567 (79%)	84%	53%
25-30	19773 (13%)	79%	50%
30+	4288 (3%)	70%	44%
Eligibility for military duty			
Eligible (*)	152312 (98%)	84%	52%
Various degrees of issues	3540 (2%)	67%	30%
District unemployment rate (%)	2.8 (1.4)		

Levels marked with (*) are used as reference group in the multivariate model

**Fig. 5:**
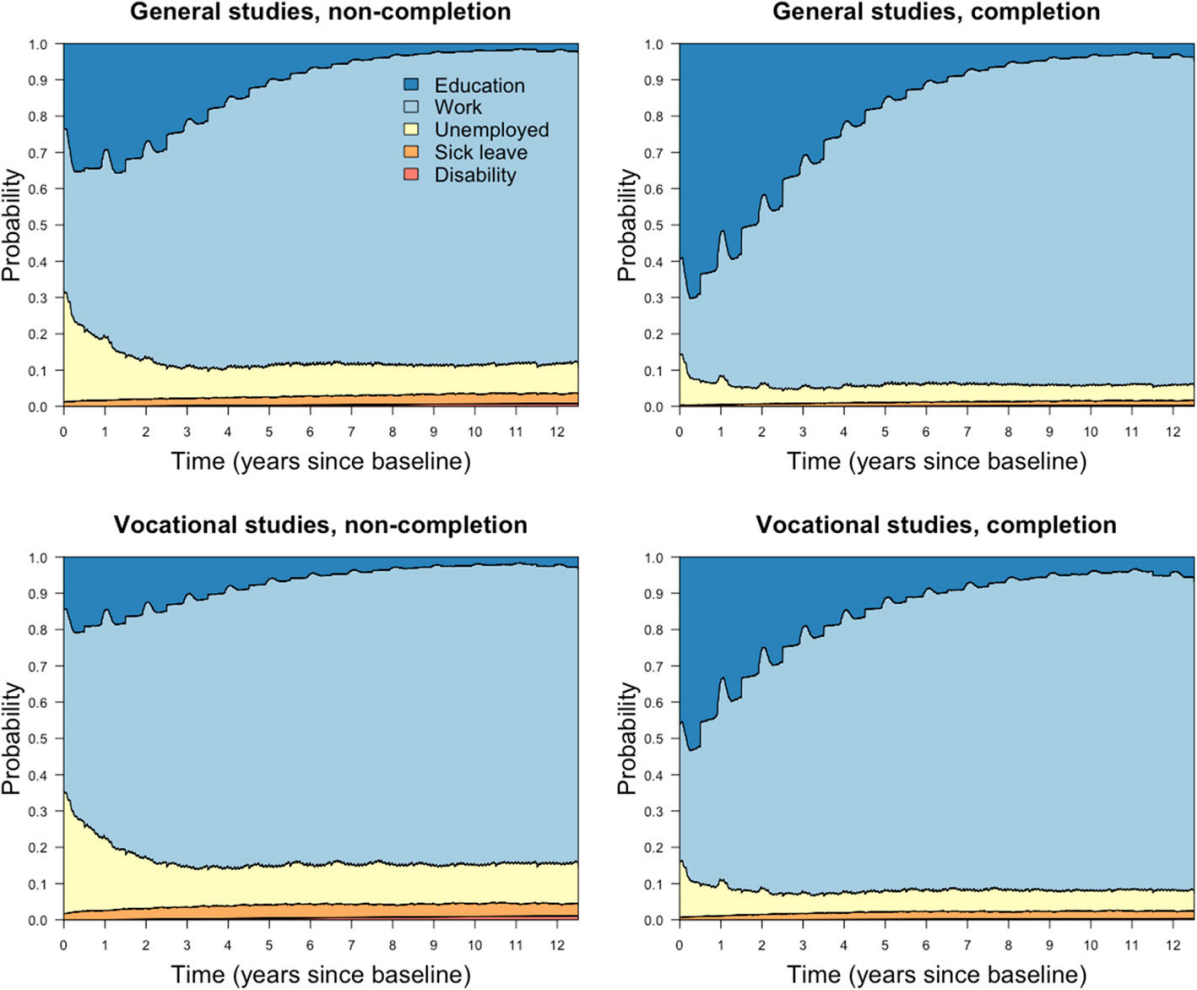
Cox regression. State occupation probabilities, favourable covariates

The state probabilities with favourable covariate values are displayed in Fig. 5. Both completers and non-completers of both fields are better off than in the unadjusted analysis (Fig. 3). Now, general studies are associated with less unemployment, sick leave and disability compared to vocational tracks. Furthermore, the probability of education is high for these covariate values regardless of exposure. In both examples (Figs. 4 and 5), non-completers and completers differ less in terms of education than in Fig. 3. This was also reflected in the Cox regressions, with the exposure effects on transitions into education decreasing considerably when adjusting for covariates.

### Inverse probability weighting

**Fig. 6:**
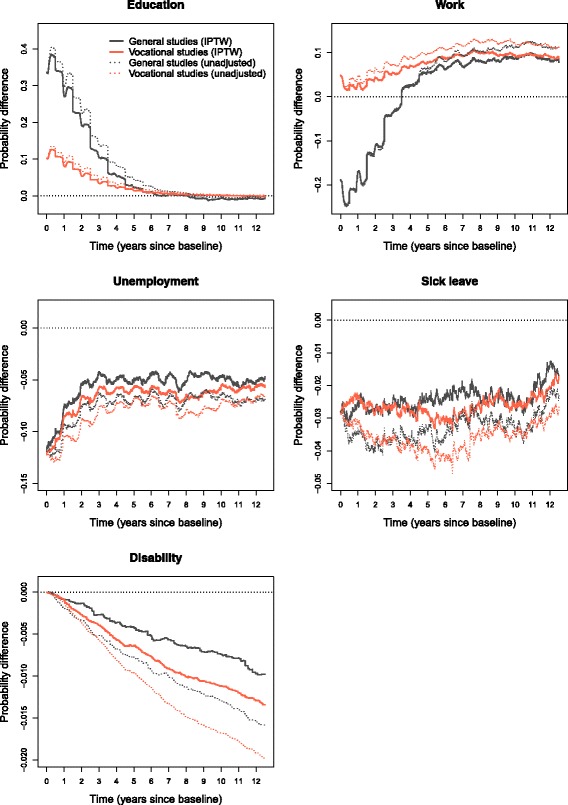
Inverse probability weights, absolute effects of completion. State probability differences between completion and non-completion (reference non-completion), the zero line indicates no difference

**Fig. 7:**
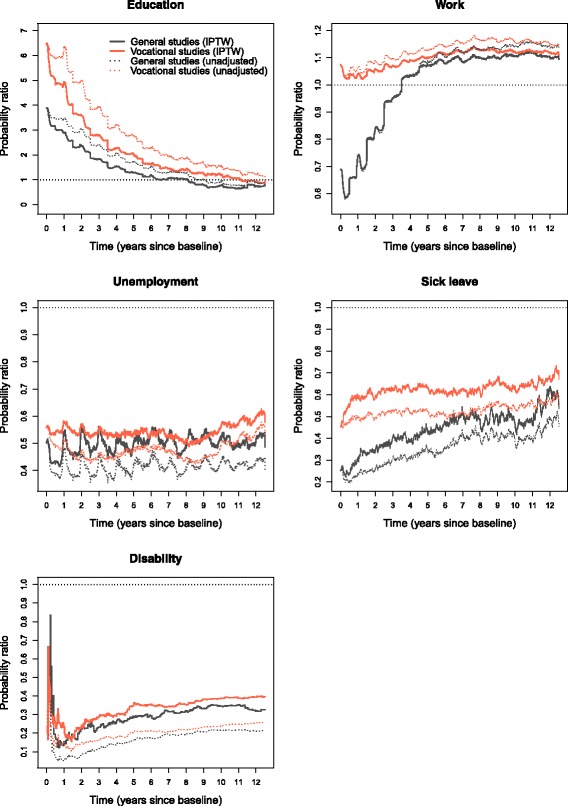
Inverse probability weights, relative effects of completion. State probability ratios between completion and non-completion (reference non-completion), the horizontal line of value 1 indicates equal probability

As previously described, we fitted logistic regression models to estimate inverse probability of treatment weights. Using the weights, we calculated transition specific weighted Nelson-Aalen estimates of the transition hazards, which were plugged into the matrix product formula for state probabilities. We chose not to plot the state probabilities using the IPTW approach as there were only minor changes in state probabilities, compared to Fig. 3, which made it hard to appreciate the consequences of weighting. These appear more clearly when plotting probability differences between completion and non-completion (absolute effect of completion; reference non-completion) shown in Fig. 6. Here we have included estimates from the weighted analyses (full-drawn lines) and also the unadjusted analyses (dotted lines). Probability ratios (relative effect of completion) are included in Fig. 7.

In Fig. 6, we find that completing general studies increases the probability of being in education the first year of follow-up by 35–40 percentage points (pp) compared to non-completion. Six years into follow-up, completers and non-completers have equal probability of education. This implies that for six years, accumulated educational attainment in terms of expected mean educational years is diverging between completers and non-completers, but the rate of divergence is declining. After six years, non-completers in the general track have a slightly higher probability of being under education. The absolute effect of completion is smaller within vocational tracks; completion leads to around 10 pp higher probability of education the first year. Despite this, the relative effect of completion is largest for vocational tracks as we see in Fig. 7.

The probability of being in work is lower for completers of general studies than all other groups the first three and a half years, which is seen in connection with the higher probability of being under education in this period. Initially, the probability of work is reduced by around 20 pp. From three and a half years into follow-up, completion increases the probability of employment and the effect fluctuates around 8-9 pp increased probability the following years. In vocational tracks, completion increases the probability of work the entire follow-up period, the first year by a couple pp, then 9-10 pp the last five years.

Completion in both fields reduces unemployment the whole period, but the absolute effect diminishes (Fig. 6). The effect is highest the first two years, with 6-12 pp lower probability of unemployment. After this, the effect fluctuates around seemingly stable levels: 6 pp reduction for vocational tracks; 5 pp for general studies. However, we find that the relative effect of completion on unemployment is more stable over time by considering the probability ratios (Fig. 7). On a relative scale, completion has greater effect in general studies, but fluctuates up and down more frequently. Nevertheless, both lines are close to 0.5 for most of the period, meaning that the probability of unemployment is roughly 50 percent of that of non-completers.

For both fields, the lines illustrating the effect of completion on sick leave (Fig. 6) seesaw rapidly around a reduction of 2-3 pp for many years, but move closer to 1.5 pp reduction towards the end. Closer inspection of state probabilities reveals that it is for non-completers sick leave is reduced towards end of follow-up. Also on a relative scale (Fig. 7), the effect diminishes, and as was the case with unemployment, the effect is greatest from completion of general studies.

By the end of follow-up, completers in general studies had around 1 pp lower probability of having transferred to permanent disability pension, compared to non-completers; in vocational tracks, disability pension was lowered by 1.25 pp. On a relative scale, disregarding the first year with very few events, general studies completers had between 1/5 – 1/3 the probability of disability compared to non-completers, while completion in vocational tracks resulted in 1/5 – 2/5 the probability of disability of non-completers. As we can see in Fig. 7, the relative effect is diminishing slowly. Thus, the absolute effect is increasing and the relative effect diminishes, which simply means that non-completers have a higher rate into disability than completers, but that the rates differ gradually less as time passes. A closer inspection reveals that for non-completers rates are slowly declining, from being high in the beginning, while for completers, rates are lowest the first few years before increasing somewhat.

**Fig. 8:**
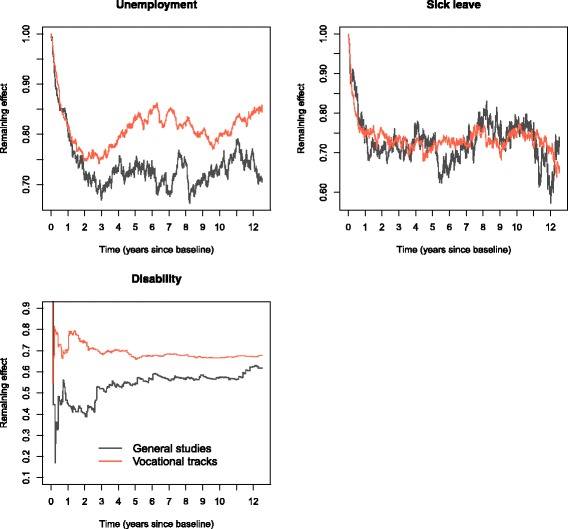
Remaining effects in IPTW analyses compared to unadjusted effects. Ratio between weighted probability differences and unadjusted probability differences

Contrasting weighted and unadjusted curves is useful to get a sense of how weights affect the results. In Fig. 8, the ratios of weighted and unadjusted probability differences are displayed for unemployment, sick leave and disability. The plots suggest that weighting reduces the effect of completion for these outcomes slightly more in general studies than for vocational tracks.

## Discussion

Our results show that, in both fields of upper secondary education, completers do better on a whole range of outcomes compared to non-completers. However, the effects change over time. For general studies, the effect of completion on education and employment changes dramatically over time and appears to be quite complementary; a large proportion of completers goes on to further education early, before transferring to work. Vocational students have higher probability of early employment compared to students from general studies – this goes for both non-completers and completers. Despite the gains in early employment, vocational students have the lowest probability of work towards the end of follow-up and generally the highest probability of unemployment, sick leave and disability. The exception was when we considered low IQ and parental SEP, where vocational track students were better off compared to general studies. Across all groups, unemployment is most common the first 2-3 years. In this period, the absolute effect of completion on unemployment diminishes, while the relative effect remains more stable over long terms. All groups experience an increase in sick leave the first few years, but the effect of completion on sick leave gets smaller over time. In the probability difference plots (Fig. 6), we saw that non-completers are more likely to have transferred to disability as time passes compared to completers; still, on a relative scale, the effect of completion diminishes. A closer inspection reveals that, even though the rate into disability remains highest for non-completers, it is slightly declining, while it is slightly increasing for completers.

The results suggest that non-completers have an added disadvantage when applying for jobs at young age, but could also mean they are not as active in seeking jobs. Furthermore, non-completers seem to have less secure jobs later on. The effect of completing vocational tracks, with regards to work, does not appear to be significant before after a few years into follow-up, which could suggest that some non-completers leave vocational studies due to employment possibilities. Sick leave increases during follow-up, which is likely due to more people in employment having obtained the right to paid sick leave. Non-completers have more sick leave and disability compared to completers across all groups, which could mean their jobs are more demanding on health, but could also be due to differences in lifestyles and health habits. Among non-completers, a reduction in sick leave is observed towards end of follow-up, which could indicate that non-completers are “closing the gap” – in terms of either education or experience – leading to better jobs and health, but it could also mean that some of the least healthy have transferred to disability pension. Disability is by definition an absorbing state, so people cannot return once entered. That the rate into disability is slightly declining for non-completers, but slightly increasing for completers, suggests that among non-completers there are individuals with an elevated risk of disability, making them transfer quickly.

The gain in early employment was offset by less tertiary education and more unemployment, sick leave and disability for vocational tracks. This could imply that vocational students are easy to employ, but less adaptable to changes in the labour market and that typical vocational trades are more taxing on health. However, it could also be related to unmeasured factors making vocational and general students different in other ways. The absolute effects of completion on unemployment, sick leave and disability are somewhat larger for vocational tracks than for general studies, and because more people attend vocational tracks, and non-completion is more prevalent, non-completion here is a bigger public concern than in general studies. However, the relative effects of completion on these outcomes are larger for general studies, which suggests that non-completers here are at greater disadvantage relative to completers, compared to in vocational tracks. The contrasts between weighted and unweighted results could indicate that more of the associations between exposure and outcomes are explained by other covariates in general studies compared to vocational tracks.

There can be several reasons to why completion leads to differentiation on the labour market. The most obvious would be that non-completers, given their status, lack a diploma – a proof of completion. Often when applying for jobs, even jobs that require no formal skills, an employer will ask for applicants’ diploma from upper secondary education. Other than simply lacking a diploma, non-completers were in some way unable to complete the requirements for completion; this could mean that grades were too low, lack of attendance or missing compulsory work. Thus, completers may have required skills useful both in a professional or educational environment, but also at a personal level making them better equipped for getting and keeping a job. Furthermore, non-completion could be connected to a feeling of failure for the individuals concerned, which potentially could lead to lower self-esteem and stigmatizing. Completion can therefore be seen as a combination of factors in addition to having a diploma.

Our measured confounders include detailed information on family background and cognitive ability (IQ). The individual health variables (BMI, military duty eligibility (mental and physical health check) and childhood chronic disease) are slightly more crude measures. An example of a potential unmeasured confounder would be that we have no clear indication of a person’s motivation to succeed in education and jobs. However, we have a young cohort, and have excluded those who did not complete conscript examinations or did not start upper secondary education. This removes individuals with the most severe health issues, making the study population more homogeneous, reducing the chance for residual confounding. Even though certain known confounders are not measured directly, the “sum” of the covariates we have adjusted for, may sufficiently reduce bias when comparing exposures. In the analyses, we only adjust for variables measured prior to the exposure. The only exception is military conscript data, which may be measured during upper secondary education (at age 18). However, regarding IQ scores, there are studies indicating that cognitive ability assessed by typical IQ tests show substantial stability from childhood to later life [41, 42].

Direct comparisons with previous studies are difficult, as there are no similar types of analyses on this topic. Several studies have looked at the consequences of non-completion, for example [2, 3, 5–7], but these are mostly cross-sectional studies, often considering only a few non-recurring outcomes. In addition, most do not analyse the two fields of education separately. Some of the broad implications in these studies are more or less the same as from our analyses; non-completers are at increased risk of receiving various types of medical- and non-medical benefits [2, 3, 5, 7]. In the paper by de Ridder (2013) [3], they studied the risk of long-term sickness absence and disability pension from age 24–29 after dropout from upper secondary education, while controlling for health variables, school problems and parental SEP. They found a crude risk difference for dropout of 21 pp, and an adjusted risk difference of 15 pp. The absolute numbers are incomparable to our results because of the time aspect, but the remaining risk difference (15/21≈0.7) is in line with the impact of weighting in our analyses. Falch (2010) [7] studies how completion affects the probability of 1) being a job seeker, 2) receiving welfare benefits, 3) being in education and 4) going to jail, during autumn (September - December) 5 years after starting upper secondary education. They approach the issue of confounding in two ways. The first is by multiple regression controlling for factors affecting completion (grades in lower secondary education, gender, 1. or 2. generation immigrant, parental education, chronic disease in childhood, distance to schools, unemployment rate, regional factors, type of education). In their results, quantitative effects are reduced by 35–70 percent compared to a crude analysis, which is comparable to our results from the weighted analysis with remaining effects typically between 40–85 percent. The second method they use to account for covariates, is to compare “equal groups”, where completion or non-completion may have happened “by chance”. More specifically, they compare students that “barely” completed to students who were very close to completing. A weakness of the study is that they consider only a short time-window of 4 months. Our study is unique in that it includes multiple recurring end-points over a long follow-up period in a large cohort, allowing us to see time-varying effects, while at the same time adjusting for several important confounders including family background, prior health and cognitive ability.

In our analyses, we only looked at the effect of a baseline exposure, and from the current analyses it is difficult to assess the effect of state histories on future outcomes. Other interesting questions could, for instance, be how tertiary education early on, or a high number of sick leave days or long periods of unemployment, affect later state probabilities. Another interesting approach would be to study the trajectories of individuals that end up in certain states, e.g. disability. This would call for even more advanced methods. A possible extension for future research, is to expand the state-space of the multi-state model. For instance, we could include more than one form of sick leave, e.g. based on diagnoses. Similarly, we could separate between high and low income work, based on taxable income, which would let us study differences in occupational social position in addition to work-participation.

Note that there is a fairly high percentage starting in vocational tracks in Norway compared to other countries. There are also very few that choose not to enrol in upper secondary education studies, even among individuals planning careers in untrained professions. Also, there are high rates of sick leave and disability, and relatively low unemployment rates in Norway. Hence, all our results might not be generalizable to other countries. Many vocational tracks professions in Norway also require a diploma of craftsmanship, which is not the case for many other countries. Thus, negative employment outcomes for vocational track could potentially be more frequent compared to other countries. Moreover, contrary to many other countries, entry into tertiary education (universities/colleges) is not contingent upon having graduated from upper secondary education as there are many ways of obtaining admission rights.

## Conclusion

The results suggest that completing upper secondary education increases long-term work participation and lowers health-related absence for young men, but that effects of completion diminish over time. Studies that have used shorter follow-up periods could be overstating the negative effects of dropout on labour market participation. We are however unable to differentiate between occupational social positions in our model. Multi-state models are well suited to analyse data on work participation and health-related absence, as they allow us to look at effects over time for multiple outcomes simultaneously. By including long histories of follow-up data, we do not have to choose between possibly arbitrary end-points that may affect the results. It is our opinion that when studying multiple, and possibly recurrent, time-to-event outcomes on education, work and health, multi-state analyses have great benefits.

## Abbreviations

HR: Hazard ratio
IPTW: Inverse probability of treatment weighting
pp: Percentage point
SEP: Socioeconomic position
